# Unveiling the ambient-condition structure of Sr_2_FeIrO_6_: a triclinic phase through synchrotron-based X-ray techniques and high pressure

**DOI:** 10.1107/S2052252525008218

**Published:** 2025-10-13

**Authors:** Samuel Gallego-Parra, Hussien Helmy Hassan Osman, Virginia Monteseguro, Catalin Popescu, Javier Ruiz-Fuertes, Paula Kayser, Vanesa Paula Cuenca-Gotor, Tania María García-Sánchez, Francisco Javier Manjón, Julio Pellicer-Porres, Gastón Garbarino, Juan Ángel Sans

**Affiliations:** a Instituto de Diseño para la Fabricación y Producción Automatizada, MALTA Consolider Team Universitat Politècnica de València 46022València Spain; b European Synchrotron Radiation Facility 38043Grenoble France; c Departamento de Física Aplicada-ICMUV, MALTA Consolider Team Universitat de València Dr. Moliner 50 Burjassot 46100Valencia Spain; d Departamento CITIMAC Universidad de Cantabria Avda. Los Castros 48 39005Santander Spain; e CELLS – ALBA Synchrotron E-08290 Cerdanyola del Vallès Barcelona Spain; f Instituto de Ciencia de Materiales de Madrid, CSIC C/Sor Juana Inés de la Cruz 3 28049Madrid Spain; Australian Nuclear Science and Technology Organisation and University of Wollongong, Australia

**Keywords:** Sr_2_FeIrO_6_, double perovskites, structural characterization, materials science, high-pressure powder diffraction, inorganic chemistry

## Abstract

Double perovskites with the formula *A*_2_*BB*′O_6_, such as Sr_2_FeIrO_6_, are being explored as unique platforms for unveiling diverse structural, electronic and magnetic properties. Inclusion of 3*d* and 5*d* transition metals in the *B* and *B*′ sites, as in Sr_2_FeIrO_6_, enables the study of electron correlation and spin–orbit coupling effects to tailor promising properties. The structure of Sr_2_FeIrO_6_ under ambient conditions has been widely debated, with theoretical and experimental studies suggesting several potential structures. This uncertainty complicates the understanding of its magnetic and electronic behaviours. High-pressure X-ray diffraction measurements, complemented by high-resolution powder X-ray diffraction studies under ambient conditions, have provided a definitive structural characterization of Sr_2_FeIrO_6_, crucial for interpreting its properties and guiding future research.

## Abstract 2

The room-temperature structure of Sr_2_FeIrO_6_, a double perovskite with notable magnetic and structural properties, has been debated extensively. Our high-resolution and high-pressure synchrotron-based powder X-ray diffraction findings provide compelling evidence to reconcile these divergent positions, laying groundwork for future research.

## 1. Introduction

Double perovskites with the formula *A*_2_*BB*′O_6_ have garnered significant interest due to the capability to accommodate different elements, with varying oxidation states, in *A*, *B* and *B*′ sites. This flexibility enables the design of materials with diverse structural, electronic and magnetic properties, and showing emerging exotic phenomena such as tunnelling-type magnetoresistance in Sr_2_FeMoO_6_ (Kobayashi *et al.*, 1998 ▸), half metallicity in *A*_2_CrWO_6_ (*A* = Ca, Sr, Ba) (Philipp *et al.*, 2003 ▸), the ferromagnetic insulator state in Y_2_CoMnO_6_ (Das & Choudhary, 2021 ▸), ferrimagnetism in *A*_2_CrOsO_6_ (*A* = Sr, Ca) (Morrow *et al.*, 2016 ▸), the magneto-caloric effect in Nd_2_*B*MnO_6_ (*B* = Co, Ni) (Li *et al.*, 2021 ▸) and the spin-glass state in Sr_2_FeCoO_6_ (Pradheesh *et al.*, 2012*a* ▸; Pradheesh *et al.*, 2012*b* ▸). These features emerge through the interplay of 3*d* and 5*d* transition metals in *B* and *B*′ sites. Among these, Sr_2_FeIrO_6_ is particularly noteworthy due to the intriguing magnetic and structural phenomena arising from the combination of Fe and Ir, driven by strong spin–orbit coupling (SOC) and electron correlation effects.

The structure of *A*_2_*BB*′O_6_ double perovskites has been the subject of numerous studies, where the mismatch in the sizes of the cations is revealed to be a crucial factor, evaluated through the Goldschmidt tolerance factor, *t* (Goldschmidt, 1926 ▸):
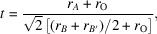
where the *r_i_*’s represent the ionic radii of *A*, *B*, *B*′ and O atoms. The lower the value of the mismatch, the less symmetric the structure becomes due to the high degree of tilt allowed for the *B*(*B*′)O_6_ octahedra, generally following the sequence [with their Glazer’s notation (GN)] for double perovskites (Howard *et al.*, 2003 ▸): *Fm*3*m*, *a*^0^*a*^0^*a*^0^ > *I*4/*m*, *a*^0^*a*^0^*c*^−^ > *I*2/*m*, *a*^0^*b*^−^*b*^−^ > *P*2_1_/*n*, *a*^−^*a*^−^*c*^+^ > *I*1, *a*^−^*b*^−^*c*^−^ (Kharkwal *et al.*, 2020 ▸; Vasala & Karppinen, 2015 ▸). In this particular case, Sr_2_FeIrO_6_ is a double perovskite compound with antisite disorder between Fe and Ir ions (*i.e.* occupation of Fe atoms in Ir sites and vice versa) within a distorted perovskite lattice. The structure of Sr_2_FeIrO_6_ at room temperature has been the subject of significant debate in the literature. Theoretical calculations suggest that the triclinic *I*1 structure and the monoclinic *P*2_1_/*n* or *I*2/*m* structures have very similar total energy values, making it challenging to determine the most stable structure solely through computational means (Roy & Kanungo, 2022 ▸). Experimental studies have also demonstrated uncertainty regarding the room-temperature structure, with some prioritizing monoclinic structures (Qasim *et al.*, 2013 ▸; Bufaiçal *et al.*, 2016 ▸; Laguna-Marco *et al.*, 2015 ▸; Retuerto *et al.*, 2021 ▸) whereas other studies have suggested that the most stable structure is triclinic under ambient conditions (Battle *et al.*, 1999 ▸; Kharkwal & Pramanik, 2018 ▸). Resolving this structural ambiguity is crucial for understanding the material’s properties and aligning scientific findings in the literature.

A review of the existing literature reveals that a triclinic phase in double perovskites, with the GN *a*^−^*b*^−^*c^−^* (different out-of-phase tilt angles along the *a*, *b* and *c* axes), is relatively uncommon and has primarily been observed in systems like Sr_2_*B*MoO_6_ (where *B* = Fe, Co, Ni, Zn, Ti, Mg) (Vasala *et al.*, 2010 ▸; Alarcon *et al.*, 2012 ▸; Marrero-López *et al.*, 2009 ▸). Conversely, for Sr_2_*B*IrO_6_ compounds, a monoclinic *P*2_1_/*n* phase, with the GN *a*^−^*a*^−^*c^+^* (same out-of-phase tilt angles along the *a* and *b* axes, different from an in-phase tilt angle along the *c* axis) predominates particularly for *B* = Ce, Ca, In, Sc, Lu, Ni, Tb and Y (Harada *et al.*, 2000 ▸; Jung & Demazeau, 1995 ▸; Kayser *et al.*, 2015 ▸; Wakeshima *et al.*, 1999 ▸; Kayser *et al.*, 2013 ▸; Zhou *et al.*, 2005 ▸; Ranjbar *et al.*, 2015 ▸). In double perovskites where one of the *B* sites is fixed as Fe and the secondary cation is varied, such as in Sr_2_Fe*B*O_6_, a tetragonal *I*4/*m* phase, with the GN *a*^0^*a*^0^*c*^−^ (no tilt along the *a* and *b* axes and an out-of-phase tilt along the *c* axis), is often found, especially for *B* = Co, Re, Nb, Sb and Os (Pradheesh *et al.*, 2012*a* ▸; Pradheesh *et al.*, 2012*b* ▸; Nakamura & Oikawa, 2003 ▸; Rosas *et al.*, 2019 ▸; Faik *et al.*, 2010 ▸; Retuerto *et al.*, 2009 ▸). These patterns hint at a possible dependence of the symmetry of the structure on the nature of both *B* and *B*′ ions, further complicating the structural determination of Sr_2_FeIrO_6_.

The exact structure of Sr_2_FeIrO_6_ under ambient conditions has been contested, leading to differing interpretations and reported properties. Previous studies have used various experimental techniques, such as X-ray diffraction (XRD) and neutron diffraction, to propose different structural models. Theoretical studies add complexity by indicating that multiple structures are energetically comparable. This hinders a comprehensive understanding of the material’s magnetic and electronic behaviours, which are highly dependent on the precise arrangement of atoms in the crystal lattice.

Here, we address and resolve this controversy through synchrotron-based studies utilizing high-resolution powder X-ray diffraction (HR-PXRD) and high-pressure powder X-ray diffraction (HP-PXRD). Our synchrotron-based investigations provide a more accurate and definitive structural characterization of Sr_2_FeIrO_6_ than previous works. Through HR-PXRD and HP-PXRD techniques, we can distinguish between the competing structural models and establish the most accurate description of the crystal structure at room temperature. This resolution of the structural ambiguity is pivotal for accurately interpreting the physical properties of the material and for guiding future research and applications involving Sr_2_FeIrO_6_ and its related double perovskites.

## 2. Experimental

### 2.1. Sample preparation

A polycrystalline sample of Sr_2_FeIrO_6_ was prepared by wet chemistry methods followed by annealing treatments. Stoichiometric amounts of Sr(NO_3_)_2_ (99%), IrO_2_ (99.9%) and FeC_2_O_4_·2H_2_O (99%) were dissolved in an aqueous solution of citric acid (10% *w*/*w*) and 1 ml of nitric acid to facilitate the dissolution of the starting materials. IrO_2_ was not dissolved in the solution but remained in suspension under magnetic stirring. The resulting suspension was gently heated until the organic resins formed, ensuring a homogeneous distribution of the involved cations. After evaporation, the resins were dried at 140°C and then heated at 600°C for 12 h (with a heating rate of 2°C min^−1^) to decompose the organic materials and eliminate the nitrates. This treatment produced highly reactive precursor materials, which were then heated in air at 1100°C for 12 h to obtain a phase-pure sample.

### 2.2. High-angular-resolution X-ray diffraction

HR-PXRD experiments were performed at the powder diffraction end station of the MSPD beamline at the Spanish ALBA synchrotron, using a high-angular-resolution multianalyzer (MAD) setup. The samples were contained in 0.3 mm diameter borosilicate capillaries, which were rotated during the collection time, and transmission geometry was used. The operating wavelength was calibrated using a NIST standard silicon sample, NIST Si640D (λ = 0.4138 Å). Given the strong X-ray absorption of iridium, a 0.3 mm diameter capillary was specifically chosen to achieve a reasonable sample absorption of μ*R* = 1.1. Using the MAD setup, the analyser crystals act as additional tiny aperture slits and thus limit peak broadening induced by residual divergence of the beam impinging on the sample, by the size of the sample and the slight displacement of the sample due to wobbling. In this way, the highest angular resolution is obtained with the beamline configuration (Fauth *et al.*, 2013 ▸). The angular range covered was up to 40°.

### 2.3. High-pressure angular-dispersive X-ray diffraction

HP experiments were conducted using a membrane-type diamond-anvil cell with IIA-type diamonds, 350 µm in size. A pre-indented stainless-steel gasket with a hole diameter of 150 µm was used as a pressure chamber. We employed a 4:1 methanol–ethanol mixture as a pressure-transmitting medium, which remains quasi-hydro­static up to 10 GPa (Klotz *et al.*, 2009 ▸). Pressure measurements were performed using the Cu equation of state (Dewaele *et al.*, 2004 ▸). HP-PXRD experiments were carried out at the BL04-MSPD beamline of the ALBA-CELLS synchrotron (Fauth *et al.*, 2013 ▸), utilizing a monochromatic wavelength of 0.4642 Å and a spot size of 20 µm × 20 µm full width at half-maximum. A Rayonix SX165 CCD image plate was used to collect the diffraction patterns, which were integrated into conventional XRD patterns using *DIOPTAS* (Prescher & Prakapenka, 2015 ▸). The *GSAS-II* software (Toby & Von Dreele, 2013 ▸) was employed to perform Rietveld and Le Bail fits on powder diffractograms. Atomic positions were not refined for the high-pressure data due to limited data quality; instead, the ambient-pressure atomic coordinates were used as a fixed model for profile fitting of the high-pressure patterns. Thus, no additional atomic coordinate values are reported under high pressure. The angular range covered was up to 20°.

## 3. Theoretical calculations

First-principles electronic structure calculations were conducted using the density functional theory (DFT) framework implemented in the *Vienna Ab initio Simulation Package* (*VASP*). To avoid atomic disorder, all mixed Fe/Ir sites were modelled as idealized positions occupied exclusively by either Fe or Ir atoms, at 2*f* and 2*g* sites, respectively (Kresse & Furthmüller, 1996*a* ▸; Kresse & Furthmüller, 1996*b* ▸; Kresse & Joubert, 1999 ▸). Projected augmented wave potentials (Blöchl, 1994 ▸) were used to describe valence electrons of Sr (4*s*^2^ 4*p*^6^ 5*s*^2^), Fe (3*d*^6^ 4*s*^2^), Ir (5*d*^7^ 6*s*^2^) and O (2*s*^2^ 2*p*^4^) atoms. We used Liechtenstein’s approximation to include the on-site Coulomb (*U*) and exchange (*J*) interaction in our DFT + *U* calculations. For Fe ions, these values were fixed to *U* = 4.40 and *J* = 3.0 eV (Serrate *et al.*, 2006 ▸), and for Ir ions *U* = 2 eV (Liechtenstein *et al.*, 1995 ▸). Spin-polarized calculations were performed with the generalized gradient approximation of Perdew–Burke–Ernzerhof revised for solids (PBEsol) (Perdew *et al.*, 2008 ▸) for the exchange and correlation energy, which provided crystal structural parameters that closely match the experimental values. The unit-cell geometries and atomic positions were optimized using the conjugate-gradient algorithm (Teter *et al.*, 1989 ▸; Bylander *et al.*, 1990 ▸). Our preliminary tests, including SOC, indicated changes in lattice parameters and atomic positions of approximately 0.1% or less. Given the negligible impact and the difficulty of achieving convergence with SOC, we chose to omit SOC in this work. A plane-wave kinetic-energy cutoff of 550 eV was set, along with a dense Monkhorst–Pack grid (Monkhorst & Pack, 1976 ▸) with an 8 × 8 × 7 *k*-point sampling mesh to ensure the total energy converged to around 10^−6^ eV, with stress tensor deviations from a diagonal hydro­static form being less than 1 kbar (0.1 GPa). All the structures were visualized using the *VESTA* program (Momma & Izumi, 2011 ▸).

## 4. Results and discussion

### 4.1. Indexing

The HR-PXRD pattern of Sr_2_FeIrO_6_ (from our phase-pure, highly crystalline sample) was indexed to evaluate candidate structures reported in the literature. We tested a triclinic model (space group *I*1) against two monoclinic models (space groups *I*2/*m* and *P*2_1_/*n*) via the M20 figure of merit, developed by de Wolff (1968 ▸), with the resulting values given in Table 1 ▸. The triclinic model yielded the highest indexing quality, with a de Wolff M20 figure of merit of 172, compared with 153 for *I*2/*m* and 57 for *P*2_1_/*n*. This superior M20 value indicates that the triclinic lattice parameters provide the best fit to the observed diffraction peaks. Consistently, Le Bail profile fits for each model showed that, while all three structures could reproduce the main diffraction features, the triclinic model produced the lowest residuals and best agreement factors. In contrast, the monoclinic models showed slightly higher misfit. These results firmly point to a triclinic unit cell as the correct description of Sr_2_FeIrO_6_ under ambient conditions, even before considering Rietveld refinement.

### 4.2. Rietveld refinement under ambient conditions

**Figure 1:**
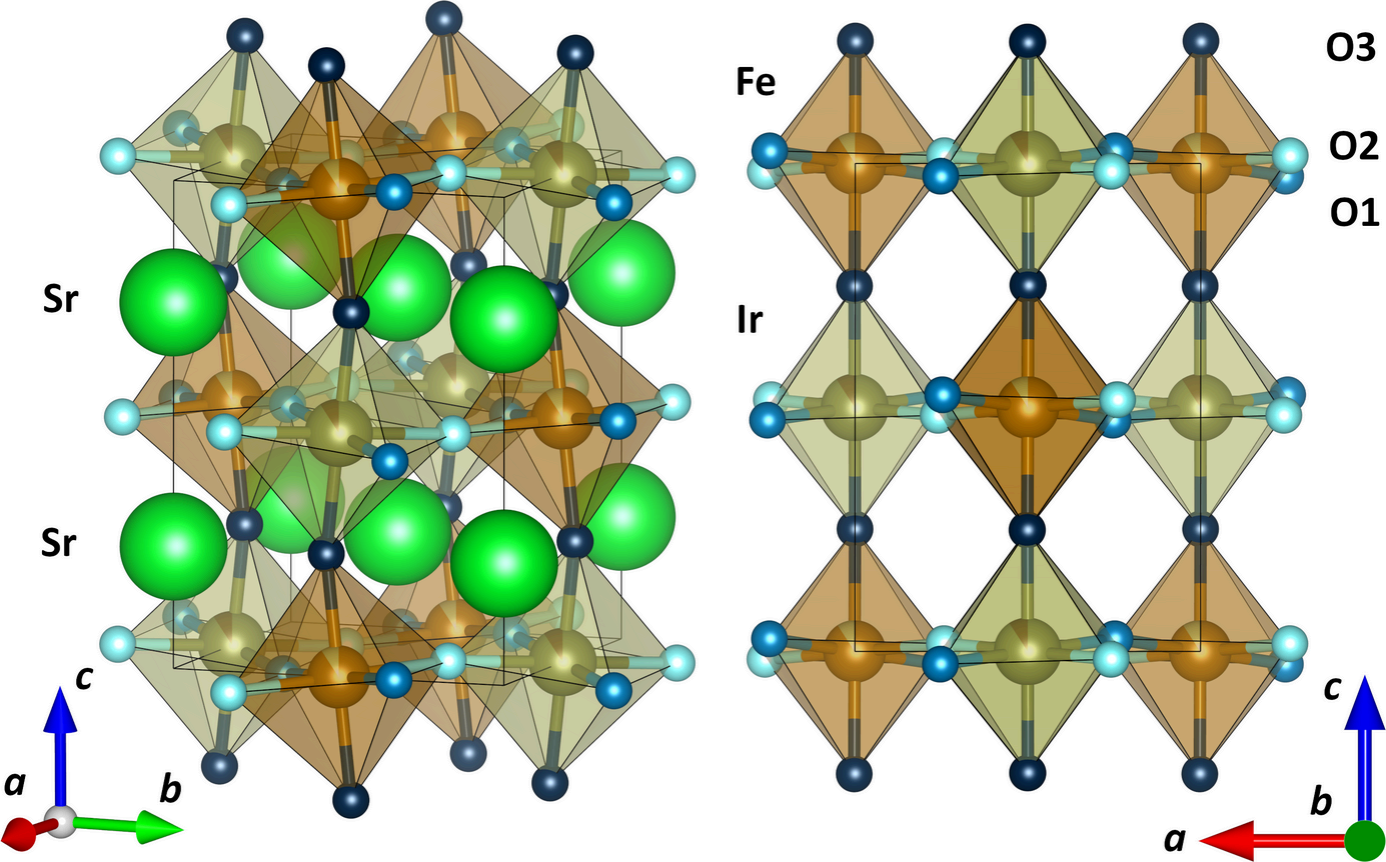
Perspective view of the triclinic structure of Sr_2_FeIrO_6_ in space group *I*1 (left panel) and the Sr_2_FeIrO_6_ structure oriented along the *b* axis (right panel). In the right panel, Sr atoms have been removed for easier visualization. One inequivalent Sr atom occupies the 4*i* sites, as do the three inequivalent O atoms. In two inequivalent positions, in 2*f* and 2*g* sites, high fractional occupancies of Fe and Ir are found, respectively. Further clarifications are given in the main text.

Rietveld refinement using the experimental HR-PXRD pattern was performed using the triclinic *I*1 model (Fig. 1 ▸). The *F*^2^_meas_ versus *F*^2^_obs_ plot from this HR-PXRD pattern is included in Fig. S1 of the supporting information. The refinement process yielded an *R*_wp_ of 13%. This relatively high *R*_wp_ value can be attributed to the complexity of the triclinic structure, which involves 25 variables: three lattice parameters, *a*, *b* and *c*; three angles, α, β and γ; three O atomic positions with the three fractional coordinates free; fractional occupancies between Fe and Ir atomic positions in 2*f* and 2*g* sites (Fig. 1 ▸, right); and eight isotropic atomic displacement parameters (see Table 2 ▸). The significant number of parameters required for an accurate fit underscores the intricate nature of the triclinic structure. The fractional occupancies obtained from the Rietveld refinement are in agreement with results obtained in the literature with values that vary between 10 and 20%. The resulting crystallographic data have been deposited at the Cambridge Crystallographic Data Centre (CCDC deposition No. 2457253).

**Figure 2:**
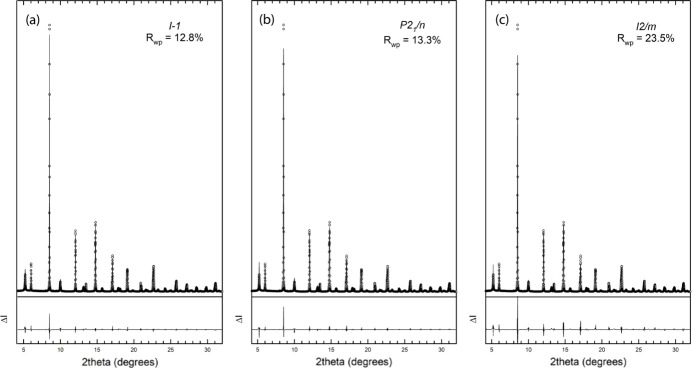
Le Bail fits of the HR-XRD pattern of Sr_2_FeIrO_6_ using an (*a*) triclinic *I*1, (*b*) monoclinic *P*2_1_/*n* and (*c*) monoclinic *I*2/*m* structure.

In addition to the Rietveld refinement, a comparative analysis of Le Bail fits was conducted to evaluate the plausibility of the three proposed structures for Sr_2_FeIrO_6_: the triclinic *I*1 structure, and both monoclinic *P*2_1_/*n* and *I*2/*m* structures, obtaining similar results to those reported by Kharkwal *et al.* (2020 ▸). This comparative analysis between the experimental XRD patterns and each of the three structures with the related residuals is depicted in Fig. 2 ▸. The residuals were found to be similar across all three fits. However, the triclinic structure exhibited the smallest residuals and the lowest quality factors, making it the most plausible structure. Despite this, the residuals for the monoclinic *P*2_1_/*n* and *I*2/*m* structures were not significantly higher, suggesting that these structures cannot be entirely ruled out. Additionally, the experimental lattice parameters and volume for the *P*2_1_/*n* and *I*2/*m* structures from Le Bail fits are given in Table 2 ▸ along with their theoretical values. To allow a direct comparison of the lattice parameters and unit-cell volume, the three space groups have been presented in their non-standard settings. Although this approach may go against certain data-validation checks performed by *checkCIF*/*PLATON* (Spek, 2009 ▸), it provides a consistent structural framework for comparison. For the triclinic model, a PLAT155 alert arises because it is not reported in its Niggli-reduced cell, whereas for the monoclinic *P*2_1_/*n* and *I*2/*m* models, a PLAT157 alert is reported since, by convention, the monoclinic angle β is required to be larger than 90º. The lattice parameters *a*, *b* and *c* of the three models agree nicely. One detail to remark on is that the β angles in the monoclinic *P*2_1_/*n* and *I*2/*m* models are predicted to be higher than 90º, unlike our experimental values, which are lower than 90º. These experimental values agree with those reported for the monoclinic *P*2_1_/*n* and *I*2/*m* models by Bufaiçal *et al.*(2016 ▸) of 89.951 (1)° and 89.72 (1)°, respectively. The present refinements highlight the inherent complexity in accurately determining the symmetry of the crystal structure of Sr_2_FeIrO_6_, providing no evidence of higher-symmetry space groups under ambient conditions. Instead, prior HR-XRD studies and our own data support the low-symmetry triclinic model (Page *et al.*, 2018 ▸). The small differences in residuals among the three proposed structures indicate that the structure under ambient conditions is not straightforward to ascertain. This ambiguity necessitates the use of new tools to resolve it.

### 4.3. High pressure behaviour

**Figure 3:**
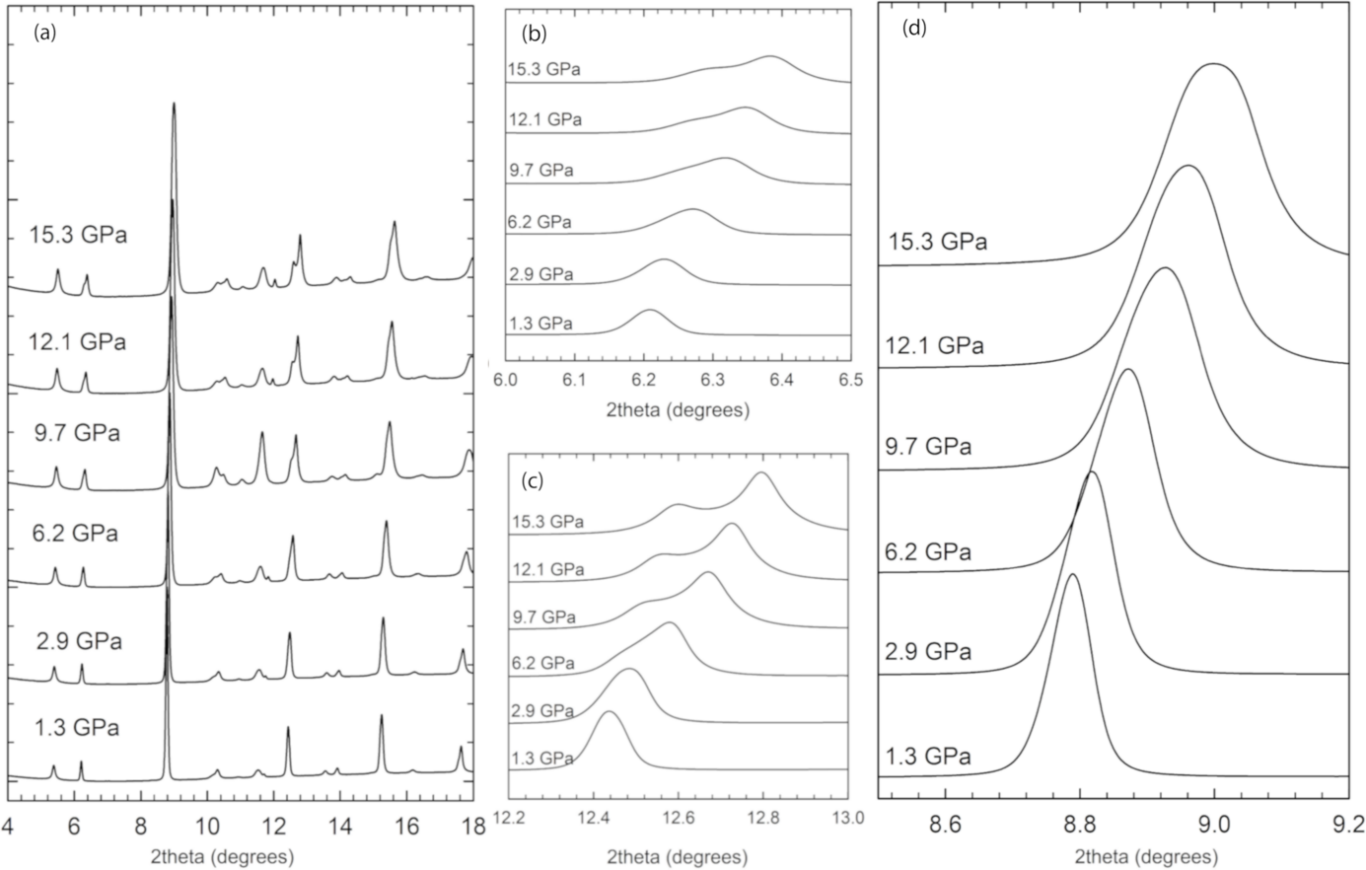
(*a*) XRD patterns of Sr_2_FeIrO_6_ at different pressures stacked and vertically shifted for clarity. HP evolution of Bragg peaks around (*b*) 6°, (*c*) 12.5° and (*d*) 8.8°.

To provide additional proof of the actual structure of Sr_2_FeIrO_6_ under ambient conditions, we performed a synchrotron-based HP-PXRD experiment [Fig. 3 ▸(*a*)]. The XRD patterns obtained under varying pressure conditions were analysed using Rietveld refinement, considering the structural factors with fixed atomic positions, *i.e.* by refining all the structural parameters except the oxygen atomic positions, due to the limitation of the 2θ range for the HP measurements. The results helped us to derive the pressure–volume equation of state and monitor the evolution of the lattice parameters and angles under high pressure. In the supporting information, Fig. S2 contains three representative 2D diffraction images at 1.4, 10 and 15.3 GPa [panels (*a*), (*c*) and (*e*)], with their respective Rietveld refinements [panels (*b*), (*d*) and (*f*)]. As can be seen, no rings from the stainless-steel gasket were observed through the whole pressure range studied, and no abrupt changes in the peak broadening above 10 GPa were seen that would indicate that the 4:1 methanol–ethanol pressure-transmitting medium had solidified.

**Figure 4:**
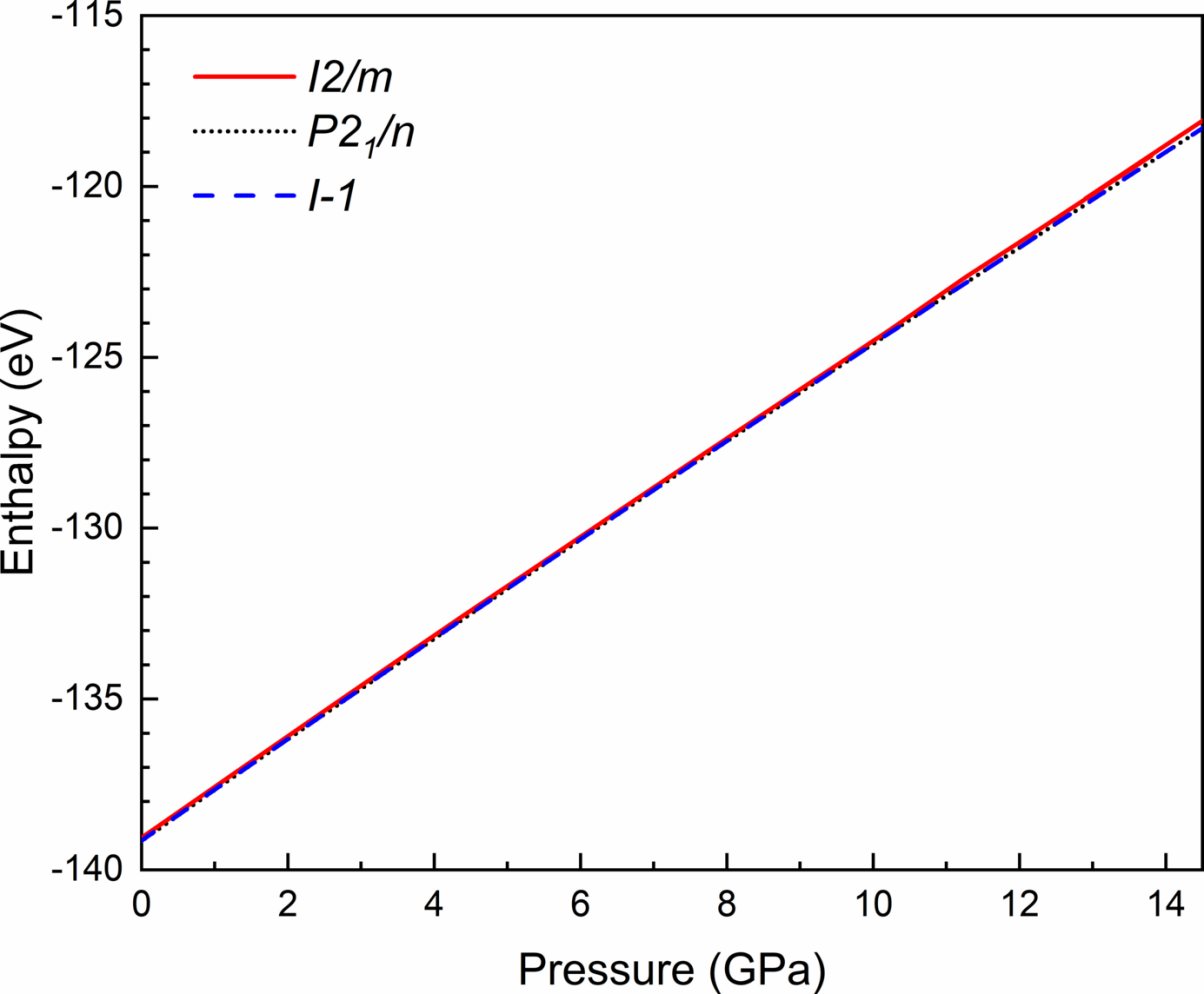
HP dependence of theoretically simulated enthalpy for the three structures proposed in the literature.

**Figure 5:**
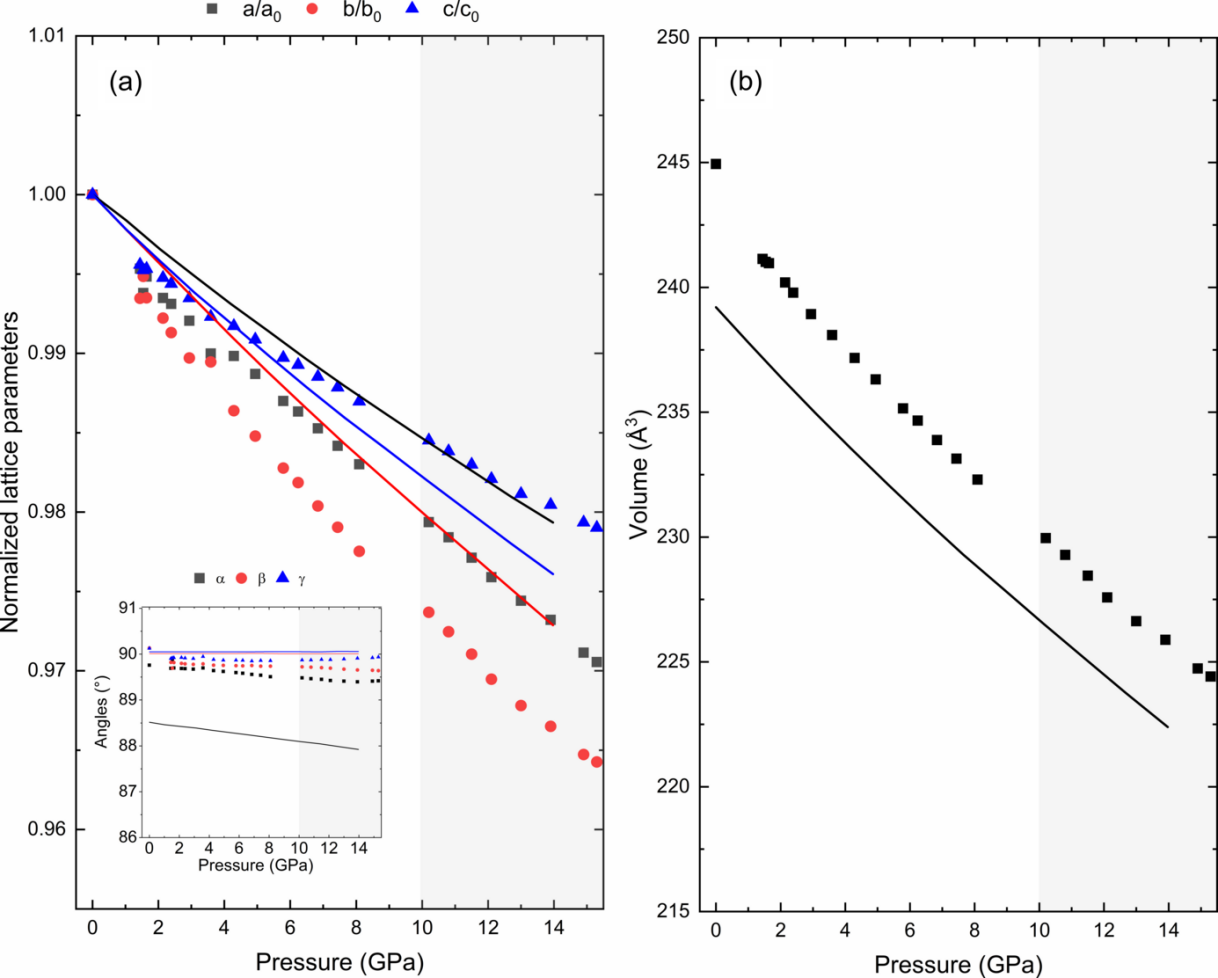
(*a*) Evolution of the normalized lattice parameters (and angles) under HP (inset) of the Sr_2_FeIrO_6_ structure in the space group *I*1, and (*b*) pressure dependence of the volume. Experimental data are represented as symbols and the theoretical calculations as solid lines. The region of quasi-hydro­static conditions up to ∼10 GPa for the 4:1 methanol–ethanol mixture pressure-transmitting medium is shaded in the plots.

During the fitting process, it became evident that the monoclinic structures were insufficient to describe the evolution of the experimental diffraction patterns throughout the entire pressure cycle. At pressures below 7 GPa, both monoclinic structures provided satisfactory fits to the diffraction patterns. However, as the pressure increased beyond 7 GPa, noticeable splits in some Bragg reflections indicated the inadequacy of these models in describing the structural changes accurately [as shown in Figs. 3 ▸(*b*), 3 ▸(*c*) and 3 ▸(*d*)]. These splits suggest the presence of additional structural complexities not captured by simpler monoclinic symmetries. Moreover, theoretical calculations for both monoclinic structures reveal no indication of a second-order phase transition, consistent with the smooth evolution of the reflection peaks. The theoretically simulated enthalpy versus pressure curves for the three proposed structures (Fig. 4 ▸) indicate that they have similar energies, with all lines overlapping within the calculation uncertainties. When the triclinic structure was employed from the beginning, the variation of lattice parameters displayed a smooth and continuous evolution with pressure, as illustrated in the experimental and theoretical normalized lattice parameters in Fig. 5 ▸(*a*). Neither the experimental nor theoretical pressure dependence of the volume show any anomaly, as depicted in Fig. 5 ▸(*b*). This critical observation indicated a more accurate representation of the actual structural changes occurring in Sr_2_FeIrO_6_. The absence of abrupt changes or anomalies in the normalized lattice parameter trends provided compelling evidence for the triclinic nature of the structure under all examined pressures. Further comparisons can be made by calculating the third-order Birch–Murnaghan equation of state (BM3-EoS) for Sr_2_FeIrO_6_, the parameters of which can be found in Table 3 ▸. The BM3-EoS was calculated using the free software *EoSfit* (Angel *et al.*, 2014 ▸).

The theoretically simulated data gave rise to a bulk modulus, *B*_0,theor_, at 0 GPa of 167.5 (1) GPa with a first pressure derivative of the bulk modulus, *B*′_0,theor_, of 4.0 (1). Experimentally, these parameters are *B*_0,exp_ = 137.1 (1) GPa and *B*′_0,exp_ = 7.1 (1). To compare *B*_0,exp_ with B_0,theor_ and the strong correlation between *B*_0_ and *B*′_0_, we fixed *B*′_0,exp_ to the value given by the theoretical calculations, attaining a *B*_0,exp_ value of 151.7 (1) GPa, showing a good agreement between both techniques.

**Figure 6:**
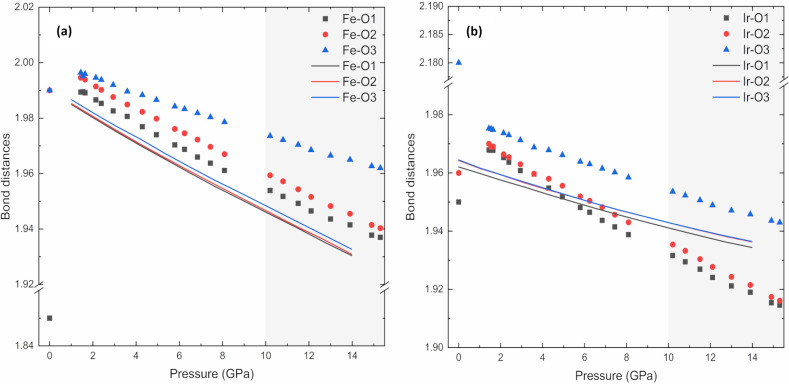
Evolution of the experimental and theoretical (*a*) Fe—O and (*b*) Ir—O bond distances under HP of the Sr_2_FeIrO_6_ structure in the space group *I*1. Experimental data are represented as symbols and the theoretical calculations as solid lines. Experimental Fe(Ir)—O bond distances represent the Fe(Ir) atoms in the atomic sites 2*f*(2*g*) with a fractional occupancy of 82.6%. The region of quasi-hydro­static conditions up to ∼10 GPa for the 4:1 methanol–ethanol mixture pressure-transmitting medium is shaded in the plots.

It is noteworthy that the smooth evolution of normalized lattice parameters for the triclinic model in the whole pressure range up to 15.3 GPa contrasts sharply with the need for a phase transition above 7 GPa when using the monoclinic models. Regarding the axial compressibilities derived from the linearized 3BM-EoS given in Table 3 ▸, the *a* and *c* axes exhibit similar pressure behaviour, both experimentally and theoretically. It is the *b* axis which, experimentally, is slightly softer as theoretically predicted but, on average, the *a* and *b* axes are more compressible than the longer *c* axis. This comparison serves as additional support for the triclinic model in the pressure range evaluated. As additional proof of a triclinic stucture being consistent with the data in the pressure range studied, we plotted the pressure evolution of the experimental and theoretical Fe—O and Ir—O distances, shown in Fig. 6 ▸. Even though the experimental bond distances from HR-PRXRD show a certain offset with respect to the high-pressure data, this does not hamper our comparison of the experimental and theoretical high-pressure trends. Experimental and theoretical Fe—O bond distances compress at a similar rate. However, this does not happen for Ir—O bond distances, which are less compressible according to the trends predicted by our theoretical calculations than the experimental trends. Regardless of this, both experimental and theoretical bond distances share a common feature: Fe(Ir)—O3 bond distances directed along the *c* axis are less compressible than the Fe(Ir)—O1, O2 bond distances located near the *ab* plane, a statement that supports a lower axial compressibility of the *c* axis than those of the *a* and *b* axes, as shown in Table 3 ▸. This can be easily explained by observing the SrO_12_ dodecahedron arrays that are placed along the *c* axis that govern its compressibility.

The necessity of introducing a phase transition in these simpler monoclinic models highlights their inability to fully capture the structural intricacies of Sr_2_FeIrO_6_ under high pressure. In contrast, the triclinic model’s capacity to explain the diffraction patterns without requiring such transitions underscores its validity. Furthermore, the continuous evolution observed in the triclinic phase model supports the hypothesis that the original structure of Sr_2_FeIrO_6_ is triclinic (Fig. 5 ▸). Additionally, attempts to index the 15.3 GPa pattern with higher symmetry cells (monoclinic *P*2_1_/*n* or *I*2/*m*) were unsuccessful – these cells could not reproduce key peak splittings and yielded significantly larger profile residuals. Neither the calculations nor the experiments provide evidence for a phase transition to a new space group up to 15.3 GPa. In particular, the smooth evolution of the lattice metrics and the absence of any abrupt anomalies suggest that Sr_2_FeIrO_6_ maintains the triclinic structure throughout this pressure range. While we cannot absolutely rule out an undetectably subtle symmetry change, our extensive analysis did not reveal any such transition. Thus, we are confident in asserting the robustness of the triclinic model in this pressure range.

The bulk modulus obtained for Sr_2_FeIrO_6_ can be contextualized by comparing it with other similar double perovskites. Sr_2_FeIrO_6_ has *B*_0,exp_ = 164 (2) GPa, which is comparable to other members of the double perovskite family, such as Sr_2_CoMoO_6_ [*B*_0,exp_ = 152 (9) GPa (Lufaso *et al.*, 2006 ▸)], Sr_2_CuWO_6_ [*B*_0,exp_ = 185 (14) GPa (Lufaso *et al.*, 2006 ▸)], Sr_2_CrReO_6_ [*B*_0,exp_ = 170 (4) GPa, *B*_0,theor_ = 172.6 GPa (Olsen *et al.*, 2009 ▸)] and Sr_2_MnSbO_6_ [*B*_0,theor_ = 157.24 GPa (Sosa-Correa *et al.*, 2023 ▸)]. This comparison indicates that Sr_2_FeIrO_6_ has experimental and theoretical *B*_0_ values that fall within the typical range for double perovskites, suggesting that its compressibility and resistance to volume change under pressure are consistent with those of similar compounds.

In summary, while both monoclinic models can describe the structure of Sr_2_FeIrO_6_ at lower pressures, they fail to account for the structural evolution at pressures beyond 7 GPa. The requirement of a phase transition above 7 GPa for these models is rendered unnecessary when a triclinic phase is considered from the outset. The smooth variation of normalized lattice parameters in the triclinic model serves as unambiguous proof of the original triclinic structure of Sr_2_FeIrO_6_, providing a more comprehensive understanding of its HP behaviour and confirming its triclinic symmetry under all examined conditions. Our results unveil that Sr_2_FeIrO_6_ maintains its low-symmetry tilt system (*a*^−^*b*^−^*c*^−^ in GN) under pressures up to 15 GPa, a finding which underscores the rigidity of this distortion. This contrasts with many perovskites where pressure induces symmetry elevation; here the triclinic distortions are persistent.

## 5. Conclusions

In this study, we have confirmed that a triclinic *I*1 crystal structure provides a more realistic model for the observed diffraction data of Sr_2_FeIrO_6_ compared with the existing models. Our synchrotron-based HR-PXRD and HP-PXRD studies provide compelling evidence that Sr_2_FeIrO_6_ adopts a triclinic *I*1 structure under ambient conditions. This conclusion is supported by the highest M20 figure of merit during the indexing process and the superior quality of Rietveld refinement achieved using the triclinic model compared with other proposed structures. The analysis also shows that the triclinic structure captures the structural complexity and atomic arrangements more accurately, thus establishing a reliable crystallographic framework for Sr_2_FeIrO_6_.

Theoretical DFT calculations further corroborate the experimental findings, revealing minimal energy differences between the proposed triclinic and monoclinic structures, yet favouring the triclinic structure based on better alignment with experimental parameters. By performing HP-PXRD we observed that the normalized lattice parameters evolve smoothly and continuously in the triclinic model, in stark contrast to the anomalies observed when using monoclinic structural models (especially above 7 GPa). Additionally, the increasing Fe(Ir)—O bond distances with pressure evolve smoothly, supporting the triclinic model. This provides robust evidence for the stability of the triclinic phase across a broad range of pressures, eliminating the need for an assumed phase transition that the monoclinic models required at higher pressures. The calculated bulk modulus of Sr_2_FeIrO_6_ at ambient pressure, derived from the pressure–volume data using a BM3-EoS, and the axial compressibilities from linearized BM3-EoS show good agreement between theoretical and experimental values. This consistency highlights the reliability of our experimental setup, and the theoretical framework used.

The findings from this research have significant implications for understanding the magnetic and electronic behaviours of Sr_2_FeIrO_6_. The precise identification of the triclinic structure clarifies the discrepancies in earlier reports. It provides a more accurate basis for exploring the material’s complex SOC and electron correlation effects, which are highly structure-dependent. This work lays the groundwork for future investigations into the exotic magnetic and electronic phenomena inherent in Sr_2_FeIrO_6_ and other related double perovskites by establishing the correct structural model. The structural insights gained here are also crucial for guiding the synthesis of these materials for technological uses where specific magnetic or electronic properties are required.

## Supplementary Material

Crystal structure: contains datablock(s) P0-Rietveld_v3-todorefinado3.bak0. DOI: 10.1107/S2052252525008218/oz5009sup1.cif

Supporting figures. DOI: 10.1107/S2052252525008218/oz5009sup2.pdf

CCDC reference: 2457253

## Figures and Tables

**Table 1:** M20 figure-of-merit values obtained when indexing the HR-PXRD pattern of Sr_2_FeIrO_6_

Model	Triclinic (*I*1)	Monoclinic (*I*2/*m*)	Monoclinic (*P*2_1_/*n*)
M20 figure of merit	172	153	57

**Table 2:** Comparison of crystallographic parameters obtained from Rietveld refinement against HR-PXRD data (‘exp’) of a model of the Sr_2_FeIrO_6_ structure in space group *I*1 (CCDC deposition No. 2457253) and parameters from theoretical (‘theor’) calculations For comparison purposes, lattice parameters and volume obtained by Le Bail fits corresponding to the space groups *P*2_1_/*n* and *I*2/*m* are given with their theoretically predicted values. *R*_wp_: weighted profile *R* factor; GOF: goodness of fit; *N*_obs_: number of observed reflections; *N*_var_: number of refined variables.

	*I*1 (exp/theor)			*P*2_1_/*n* (exp/theor)	*I*2/*m* (exp/theor)
*a* (Å)	5.5716 (3)/5.5763 (1)			5.5687 (3)/5.5450 (1)	5.5618 (2)/5.5951 (1)
*b* (Å)	5.5677 (3)/5.4790 (1)			5.5927 (3)/5.4985 (1)	5.5724 (2)/5.5384 (1)
*c* (Å)	7.8964 (2)/7.8319 (1)			7.8691 (4)/7.8621 (1)	7.8826 (3)/7.7263 (1)
α (°)	89.761 (3)/88.518 (1)			90	90
β (°)	90.134 (4)/90.013 (1)			89.8812 (6)/90.294 (1)	89.921 (4)/90.389 (1)
γ (°)	90.128 (2)/90.052 (1)			90	90
*V* (Å^3^)	244.947 (5)/239.2051 (2)			245.071 (5)/239.7069 (2)	244.303 (4)/239.4140 (2)
Sr (4*i*)	0.5	0.5	0.25		
Fe (82.6%), Ir (17.4%) (2*f*)	0	0.5	0		
Ir (82.6%), Fe (17.4%) (2*g*)	0.5	0	0		
O1 (4*i*)	0.247 (13)/0.2790 (13)	0.243 (11)/0.2757 (11)	0.992 (5)/0.9750 (5)		
O2 (4*i*)	0.244 (8)/0.2237 (8)	0.761 (9)/0.7800 (9)	0.015 (4)/0.0249 (4)		
O3 (4*i*)	0.541 (7)/0.5506 (7)	0.061 (5)/0.0016 (5)	0.271 (4)/0.2482 (4)		
*R*_wp_	13.377				
GOF	24.18				
*N*_obs_	621				
*N*_var_	17				

**Table 3:** Experimental (exp) and theoretical (theor) 3BM-EoS and axial compressibilities from linearized 3BM-EoS Experimental and theoretical pressure ranges cover up to 15.3 and 14 GPa, respectively.

BM3-EoS	Exp		Theor	Linearized BM3-EoS	Exp	Theor
*B*′_0_	7.1 (1)	4.0 (1)	4.0 (1)	*K*_a_ (10^−3^ GPa^−1^)	2.1 (1)	1.7 (1)
*B*_0_ (GPa)	137.1 (1)	151.7 (1)	167.5 (1)	*K*_b_ (10^−3^ GPa^−1^)	3.4 (1)	2.2 (1)
				*K*_c_ (10^−3^ GPa^−1^)	1.8 (1)	1.9 (1)
