# Supplementary material for: Unveiling the ambient-condition structure of Sr2FeIrO6: a triclinic phase through synchrotron-based X-ray techniques and high pressure
Source: IUCrJ. 2025 Oct 13;12(Pt 6):683–91. doi: 10.1107/S2052252525008218 (PMC12573922; doi:10.1107/S2052252525008218)
Supplement: Supplementary file 2 [file m-12-00683-sup2.pdf]

IUCrJ

Volume 12 (2025)

**Supporting information for article:**

## Unveiling the ambient-condition structure of Sr2FeIrO6: a triclinic phase through synchrotron-based X-ray techniques and high pressure

Samuel Gallego-Parra, Hussien Helmy Hassan Osman, Virginia Monteseguro, Catalin Popescu, Javier Ruiz-Fuertes, Paula Kayser, Vanesa Paula Cuenca-Gotor, Tania María García-Sánchez, Francisco Javier Manjón, Julio Pellicer-Porres, Gastón Garbarino and Juan Ángel Sans

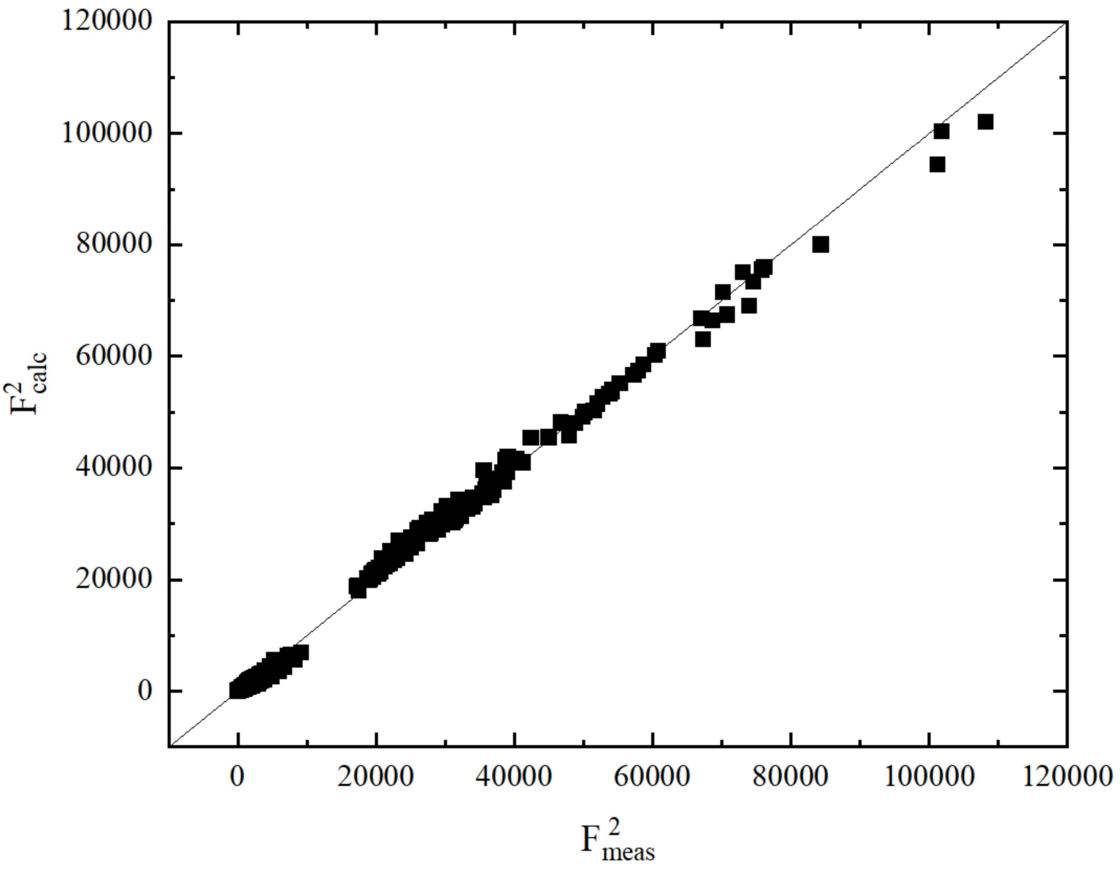


**Figure S1**. $F^2_{meas}$ vs $F^2_{obs}$ plot from HR-PXRD measurement of $Sr_2FeIrO_6$ at room conditions.

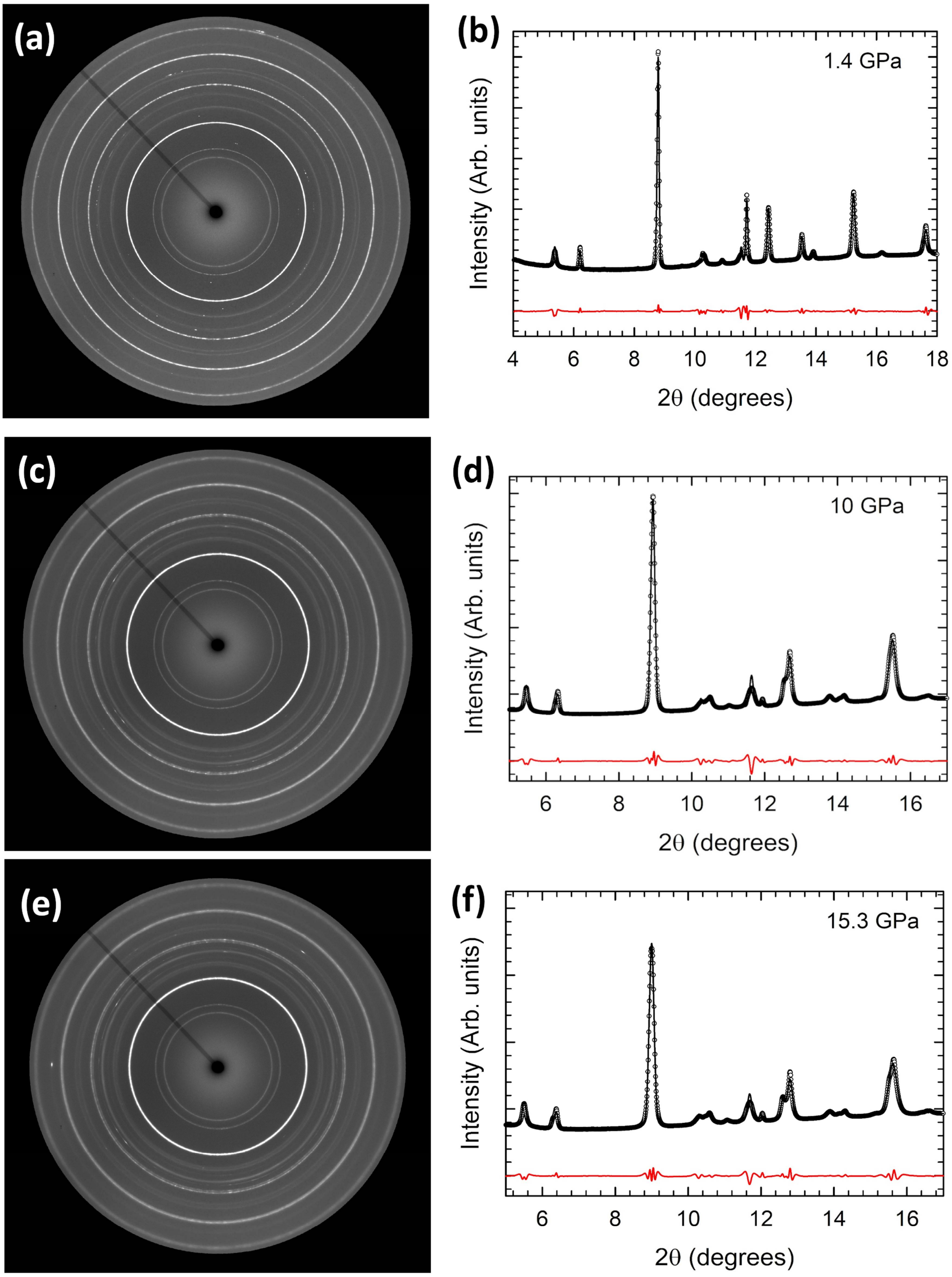


**Figure S2**. 2D images of HP-PXRD measurements at 1.4 (a), 10 (c) and 15.3 GPa (e) of $Sr_2FeIrO_6$, with their respective Rietveld refinements (b, d and f).
